# Community Outbreak Moderates the Association Between COVID-19-Related Behaviors and COVID-19 Fear Among Older People: A One-Year Longitudinal Study in Taiwan

**DOI:** 10.3389/fmed.2021.756985

**Published:** 2021-12-17

**Authors:** Yi-Jie Kuo, Yu-Pin Chen, Hsiao-Wen Wang, Chieh-hsiu Liu, Carol Strong, Mohsen Saffari, Nai-Ying Ko, Chung-Ying Lin, Mark D. Griffiths

**Affiliations:** ^1^Department of Orthopedic Surgery, Wan Fang Hospital, Taipei Medical University, Taipei, Taiwan; ^2^Department of Orthopedic Surgery, School of Medicine, College of Medicine, Taipei Medical University, Taipei, Taiwan; ^3^Department of Hydraulic and Ocean Engineering, National Cheng Kung University, Tainan, Taiwan; ^4^National Cheng Kung University Hospital, College of Medicine, National Cheng Kung University, Tainan, Taiwan; ^5^Department of Public Health, National Cheng Kung University Hospital, College of Medicine, National Cheng Kung University, Tainan, Taiwan; ^6^Health Research Center, Life Style Institute, Baqiyatallah University of Medical Sciences, Tehran, Iran; ^7^Health Education Department, Faculty of Health, Baqiyatallah University of Medical Sciences, Tehran, Iran; ^8^Department of Nursing, National Cheng Kung University Hospital, College of Medicine, National Cheng Kung University, Tainan, Taiwan; ^9^Institute of Allied Health Sciences, College of Medicine, National Cheng Kung University, Tainan, Taiwan; ^10^Department of Occupational Therapy, College of Medicine, National Cheng Kung University, Tainan, Taiwan; ^11^Biostatistics Consulting Center, National Cheng Kung University Hospital, College of Medicine, National Cheng Kung University, Tainan, Taiwan; ^12^International Gaming Research Unit, Psychology Department, Nottingham Trent University, Nottingham, United Kingdom

**Keywords:** behavior, COVID-19, fear, geriatric, psychological distress

## Abstract

Although health behavior theories indicate that fear is effective in activating preventive behaviors, the question of whether COVID-19 severity moderates the association between fear of COVID-19 and preventive behaviors remains unclear. The present study investigated the association between the fear of COVID-19 and preventive behaviors during the COVID-19 community outbreak of two severity levels in Taiwan. Data were obtained regarding the fear of COVID-19 and practice of preventive behaviors from 139 older people (mean age = 71.73 years; 30.2% men) through in-person interviews during a mild COVID-19 outbreak period (baseline assessment). Data from 126 of the 139 participants were obtained again through a telephone interview during a severe COVID-19 outbreak period (follow-up assessment). A significant increase in the fear of COVID-19 (*d* = 0.39, *p* < 0.001) and a decrease in preventive behaviors (*d* = 0.63, *p* < 0.001) were found in the follow-up assessment. The association between fear of COVID-19 and preventive behaviors was not significant at baseline (*r* = −0.07, *p* > 0.05) but became significant at the follow-up assessment (*r* = 0.32, *p* < 0.001). The severity of a COVID-19 outbreak may alter older people's psychological status and related behaviors.

## Introduction

Since late 2019 and early 2020, the increasing global spread of the novel coronavirus disease 2019 (COVID-19) has adversely affected various aspects of individuals' lives including public health, finances, and caregiving burden (1–4). Therefore, health behaviors, particularly behaviors to prevent COVID-19 infection, have become crucial for governments to efficiently control the spread of COVID-19 (5, 6). In particular, prior to the development of effective vaccines to promote herd immunity in the community (7–9), engaging in preventive behaviors is the most efficient way to reduce the transmission rate of infectious diseases including COVID-19 (10–12).

Through government policies, preventive COVID-19 behaviors can be implemented at community (i.e., authorized policies) and individual (i.e., citizens' practice) levels (11). For example, many countries have applied spatial distancing measures (e.g., “lockdowns,” quarantining) to reduce COVID-19 transmission (13–15). Such policies have demonstrated promising results in COVID-19 infection control because the infection rate was lower in countries that launched early preventive policies (e.g., Taiwan) such as border control and quarantine (11, 16, 17). Moreover, the citizens of countries that have more effectively controlled the COVID-19 pandemic exhibited more satisfactory psychological health outcomes (16). However, depending only on government measures to suppress COVID-19 infection is difficult given the longevity of the pandemic (i.e., the COVID-19 pandemic has already lasted for more than 18 months at the time of writing). Therefore, healthcare providers should identify potential factors associated with individuals' preventive behaviors at different severity levels of COVID-19 outbreak to help fight against the long-lasting COVID-19 pandemic.

The present study particularly focused on the preventive COVID-19 behaviors among older people for the following reasons. First, empirical evidence shows that compared with young adults, older people have a higher COVID-19 mortality rate (18). For instance, people older than 70 years were reported to have a COVID-19 mortality rate of 12.8% in Italy and 8.0% in China (18). The high mortality rate among older people with COVID-19 infection can be attributed to their chronic diseases such as diabetes mellitus, cancer, and cardiovascular disease (18, 19). Moreover, being overweight and obese have also been associated with more severe consequences among individuals with COVID-19 infection (14, 20). Second, because of the adverse outcomes of COVID-19 infection, older people may have poorer psychological health, including an increased incidence of stress and anxiety (21, 22). Psychological health is a crucial issue among older people because studies have reported that older people commonly have depression, anxiety, and stress, even during non-pandemic periods (23–25). Furthermore, poor psychological health among older people may cause various negative health outcomes (26, 27). On the other hand, a considerable proportion of population in any community (especially in developed countries) are elderly. Neglecting their health may threaten health of whole community and increase healthcare expenditure including the costs of hospitalization and treatment that may impose an avoidable pressure on health system. Therefore, understanding older people's preventive COVID-19 behaviors is of extreme importance because such behaviors may reduce their risk of COVID-19 infection and consequently protects them from physical and psychological health outcomes resulting after infection. Also, it prevents unnecessary stress on healthcare system as well as expensive treatment.

In the literature, three key psychological factors associated with older people's preventive COVID-19 behaviors were identified: perceived infectability, fear of COVID-19, and trust in COVID-19 information sources (28–32). Literature concerning health behavior theories such as the health belief model (29), fear drive model (30), and protection motivation theory (32) help emphasize the roles of perceived infectability and fear of COVID-19 in individuals' compliance with preventive COVID-19 behaviors. These theories indicate that perceived infectability may induce individuals' fear, leading them to engage in behaviors to cope with the fear (e.g., preventive behaviors, information searching behavior, and paying attention to COVID-19 news to cope with the fear of COVID-19). These models have been widely studied during the COVID-19 pandemic and reported to be effective in explaining a number of preventive COVID-19 behaviors (28, 33, 34). Moreover, trust in COVID-19 information may increase individuals' engagement in preventive behaviors because such information increases their awareness and instructs individuals in how to practice preventive behaviors and also contributes to understanding the severity of the problem as well as their susceptibility toward infection (35, 36).

Although perceived infectability, fear of COVID-19, and trust in COVID-19 information have been determined to be factors contributing to preventive COVID-19 behaviors (33, 34), no analytic study has examined their association during the pandemic period at different levels of COVID-19 severity. Therefore, such information can be beneficial for different stakeholders (including government policymakers and healthcare providers) to plan and implement the most effective measures for improving and maintaining preventive COVID-19 behaviors among older people. In the present study, a sample of older people residing in Taipei City, Taiwan, was recruited to examine associations among perceived infectability, fear of COVID-19, trust in COVID-19 information, and preventive COVID-19 behaviors because this sample experienced different levels of COVID-19 severity over a one-year time span.

**Figure 1:**
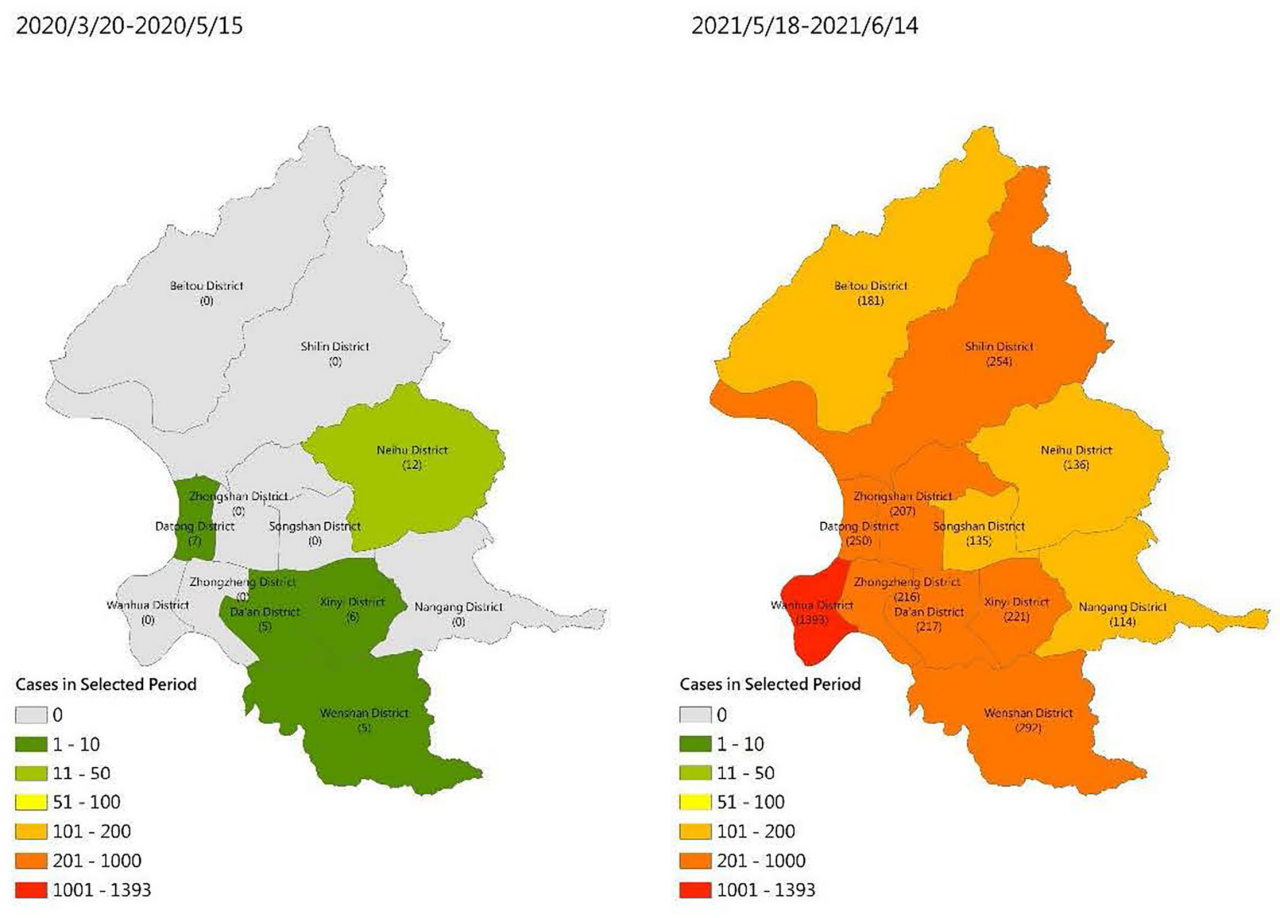
COVID-19 severity in Taipei in the time periods of two surveys (without community outbreak: from March 20, 2020, to May 15, 2020; with community outbreak: from May 18, 2021, to June 14, 2021).

The Taiwanese government applied universal policies early for controlling COVID-19 infection including providing instant information on COVID-19 through different platforms, implementing early border control, and encouraging the community to engage in preventive behaviors. This early reaction helped Taiwanese citizens have a near normal life for more than one year when other countries were experiencing considerable life changes such as lockdowns and prohibition of outdoor activities (37). However, this situation substantially changed in Taiwan, especially in Taipei City, when a severe community outbreak occurred in early May 2021. According to the Taiwan Centers for Disease Control, 441 confirmed COVID-19 cases and 7 COVID-19 deaths occurred in the period between January 1, 2020, and May 28, 2020 (https://www.cdc.gov.tw/En; accessed 28 May 2020). The number of confirmed COVID-19 cases substantially increased after May 2021: the number of confirmed COVID-19 cases was 10,846 and that of COVID-19 deaths was 438 in the period between May 18, 2021, and June 14, 2021 (https://www.cdc.gov.tw/En; accessed 15 June 2021). Moreover, on May 1, 2021, the total number of confirmed COVID-19 cases was 1,132 and that of COVID-19 deaths was 12 (https://www.cdc.gov.tw/En; accessed 1 May 2021). Figure 1 presents the different levels of COVID-19 severity in Taipei, Taiwan, during the two time periods. Moreover, Wenshan District, wherein all participants included in the present study resided, had the second most severe community outbreak among all districts in Taipei.

**Figure 2:**
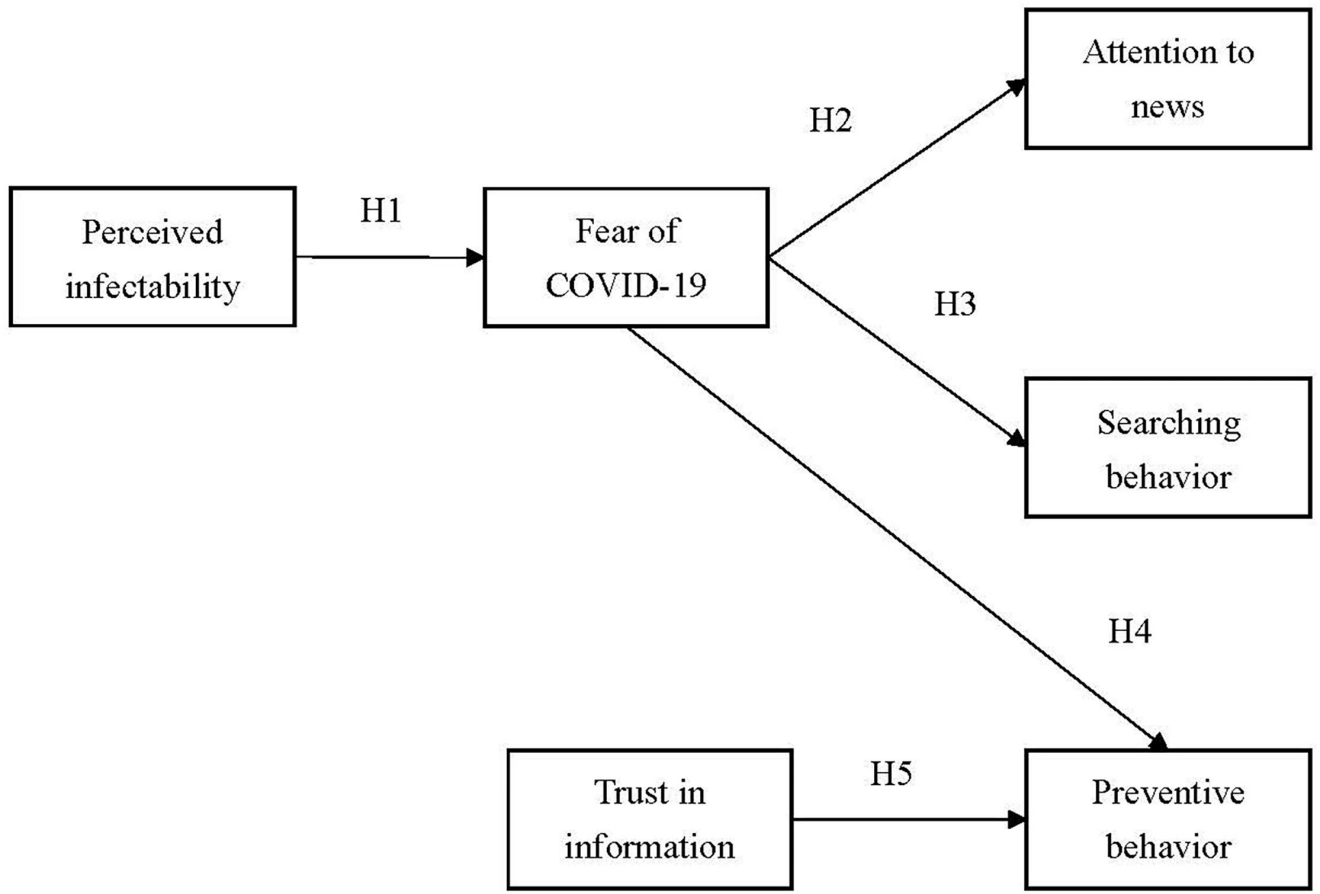
Proposed model for testing.

By conducting a baseline survey during the period with a low level of COVID-19 severity and a follow-up survey during the period with a high level of COVID-19 severity, the present study examined changes in the following factors: perceived COVID-19 infectability, fear of COVID-19, trust in COVID-19 information, paying attention to COVID-19 news, COVID-19 information searching behavior, and preventive behaviors. Moreover, the associations among these factors were hypothesized as follows (Figure 2): Hypothesis 1: Perceived infectability would be positively associated with the fear of COVID-19. Hypothesis 2: Fear of COVID-19 would be positively associated with paying attention to COVID-19 news. Hypothesis 3: Fear of COVID-19 would be positively associated with COVID-19 news searching behavior. Hypothesis 4: Fear of COVID-19 would be positively associated with preventive COVID-19 behaviors. Hypothesis 5: Trust in COVID-19 information would be positively associated with preventive COVID-19 behaviors.

## Methods

### Participants and Study Procedure

The following inclusion criteria were used at baseline to define eligible participants: (1) aged 60 years and above, (2) having the cognitive ability to understand questions used in the present study, (3) having the ability to communicate in Mandarin or Taiwanese, and (4) voluntary willingness to participate in the study. Older outpatients with a disability (e.g., severe mental illness and cognitive impairments) that prevented them from completing the present study's survey questions were excluded.

Regarding the recruitment procedure, baseline data were collected from April 20, 2020, to May 15, 2020. Follow-up data were collected from May 18, 2021, to June 14, 2021. In particular, participants were first approached at the outpatient clinic of one medical center in Wenshan District, Taipei, Taiwan, for baseline measurements. Older outpatients in the medical center were provided information regarding the present study when they visited and consulted a physician in the medical center. The aims of the present study were clearly explained to the patients before they agreed to participate in the study. All participants provided written informed consent before they began completing surveys through in-person interviews in a private room. One year later (i.e., May 2021), a sudden and aggressive community outbreak of COVID-19 occurred in Taipei (before the outbreak [December 1, 2019 to May 14, 2021]: rolling seven-day average of daily new cases = 0 to 1.6; total cases = 22; after the outbreak [May 15, 2021 to June 14, 2021]: rolling seven-day average of daily new cases = 13.7 to 230.4; total cases = 3,953; 38). Moreover, the control policy to minimize the spread of COVID-19 during the outbreak period included the following 11 points: (1) individuals engaging in outdoor activities should wear a mask and fines will be applied to those who do not wear a mask; (2) entertainment and leisure facilities should be closed and fines will be applied to those who open any entertainment and leisure facilities; (3) restaurants cannot provide dine-in services, and all meals should take-out only; (4) marriage ceremonies cannot have banquets; (5) closure of all religion facilities (e.g., temples); (6) closure of all education facilities (including kindergarten and primary schools); (7) prohibition of social gatherings of more than five people indoors and 10 people outdoors (with the exception of family members); (8) self-monitoring of health status; (9) application of spatial distancing in public agencies and business companies; (10) working from home where possible; and (11) increasing use of disinfection and sanitation in public areas and public transportation.

The participants were contacted again to complete surveys via telephone interviews by the same research team for obtaining follow-up measurements. Before the participants participated in the telephone interview, they were informed of the study purpose and their participation-related rights again. All the participants in the follow-up survey provided informed consent to participate in the study. The study was approved by the Ethical Committee of Taipei Medical University (registered number: TMU-JIRB N202005044).

### Measures

#### Demographics and Participants' Characteristics

Data were collected regarding the participants' demographic characteristics, namely age, sex, height, weight, and educational status. In addition, data were collected from participants regarding the following comorbidities: hypertension, diabetes mellitus, heart disease, renal disease, stroke, dementia, depression, and cancer.

#### COVID-19-Related Behaviors

Three types of COVID-19-related behaviors were assessed: preventive COVID-19 behaviors, COVID-19 news searching behavior, and paying attention to COVID-19 news. Three COVID-19 preventive behaviors were assessed, namely handwashing, avoiding touching the face, and covering the mouth when sneezing, on a five-point Likert scale (1 = rarely; 5 = always). Moreover, the three items shared the same item stem (i.e., “Because of the COVID-19 pandemic, …”) to clearly link the three behaviors with preventive COVID-19 behaviors. More specifically, the item descriptions were “Because of the COVID-19 pandemic, how often do you use sanitizer or soap to carefully clean your hands,” “Because of the COVID-19 pandemic, how often do you pay attention not to touch your eyes, nose, and mouth,” and “Because of the COVID-19 pandemic, how often do you use elbow or handkerchief to cover your mouth and nose when you sneeze or cough.” These three behaviors were recommended by the World Health Organization (WHO) as key preventive behaviors to avoid COVID-19 infection when the WHO first announced the pandemic (38). A higher score indicates a higher level of engagement in preventive COVID-19 behaviors. The preventive COVID-19 behavior score is computed by summing the three item scores and dividing the total by 3. To assess COVID-19 news searching behavior, a single item was used (i.e., “Have you eagerly search COVID-19 news”) that was rated on a five-point Likert scale (1 = rarely; 5 = always). To assess paying attention to COVID-19 news, a single item was used (i.e., “Have you paid attention to the COVID-19 news”) that was rated on a five-point Likert scale (1 = do not care at all; 5 = care a lot).

#### Fear of COVID-19

The seven-item Fear of COVID-19 Scale (FCV-19S) was used to assess the fear of COVID-19. All items are rated on a 5-point Likert scale (1 = strongly disagree; 5 = strongly agree). A higher score indicates a greater fear of COVID-19. The FCV-19S has been shown to have satisfactory psychometric properties across countries in different continents (16, 39) including Taiwan (28, 38). The fear of COVID-19 score is computed by summing the scores obtained for the seven items in the FCV-19S and dividing the total by 7.

#### Trust in COVID-19 Information

The six-item Believing in COVID-19 Information Scale (BCIS) was used to assess individuals' trust in COVID-19 information. All items are rated on a five-point Likert scale (1 = do not believe at all; 5 = totally believe). A higher score indicates a higher level of trust in COVID-19 information. The BCIS has been shown to have satisfactory psychometric properties in the Taiwanese population (28, 38). The score for trust in COVID-19 information is computed by summing the scores for the six items in the BCIS and dividing the total by 6.

#### Perceived Infectability

To assess perceived risk of COVID-19 infectability, a single item was used (i.e., “How large the chance that you think you will get COVID-19 infection”) that was rated on a five-point Likert scale (1 = very low; 5 = very high).

### Data Analysis

The participants' demographic characteristics were first analyzed using descriptive statistics (i.e., *means* and *frequencies*). Paired *t*-tests together with Cohen's *d* were used to examine differences in preventive COVID-19 behaviors, COVID-19 news searching behavior, paying attention to COVID-19 news, fear of COVID-19, trust in COVID-19 information, and perceived infectability between the two assessments (i.e., baseline measures before the community outbreak and one-year follow-up, which occurred after the community outbreak). Cohen's *d* was used to examine the effect of the differences between baseline and follow-up assessments, where small, moderate, and large effects were assigned values of 0.2, 0.5, and 0.8, respectively (40). Pearson's correlation coefficients were calculated to examine the bivariate association between the studied variables for the baseline and follow-up assessments, respectively.

Regarding the proposed model (Figure 2), structural equation modeling (SEM) was used to examine the fit between the proposed model and data. In particular, two SEM models were constructed; one model used the baseline data, whereas another model used the follow-up data. All the studied variables (perceived infectability, fear of COVID-19, trust in COVID-19 information, paying attention to COVID-19 news, COVID-19 news searching behavior, and preventive COVID-19 behaviors) were treated as observed variables to satisfy the principle of parsimony. Moreover, both SEM models were adjusted for age, sex, educational status, body mass index, and comorbidities. Age, sex, educational status, body mass index, and comorbidities were all controlled for because prior evidence has indicated that these factors are likely to be associated with perceived infectability or fear of COVID-19 (23, 41, 42). The SEM models were estimated using diagonally weighted least squares and evaluated using the following fit statistics: (i) non-significant χ^2^; (ii) comparative fit index (CFI) > 0.9; (iii) Tucker–Lewis index (TLI) > 0.9, (iv) root mean square error of approximation (RMSEA) <0.08; and (v) standardized root mean square residual (SRMR) <0.08 (43, 44). R software with the Lavaan package (45) was used to conduct SEM. The remaining statistical analyses were performed using IBM SPSS 24.0 (IBM Corp., Armonk, NY, USA).

## Results

Table 1 lists the participants' characteristics. A total of 139 older people (mean age = 71.73 years; 30.2% men) participated in the baseline assessment and 126 of them (mean age = 71.30 years; 30.2% men) participated in the follow-up assessment. Thirteen older people were lost to follow-up due to different reasons including death, moving, and unwillingness to participate in the follow-up assessment. However, the characteristics of 126 participants in the follow-up assessment were similar to those of the 139 participants in the baseline assessment (Table 1). Moreover, Little's missing completely at random test showed that the studied variables, namely COVID-19-related behaviors, fear of COVID-19, trust in COVID-19 information, and perceived infectability, were missing completely at random (χ^2^= 53.65, *df* = 54, *p* = 0.49).

**Table 1:** Participants' characteristics.

	***N*** **(%) or** ***Mean*** **(*****SD*****)/ range**	
	**Baseline (*n* = 139)**	**Follow-up (*n* = 126)**
Age (year)	71.73 (7.90)/60–97	71.30 (7.23)/60–88
Sex (male)	42 (30.2)	38 (30.2)
Educational status (junior high or below)	46 (33.1)	37 (29.4)
Body mass index (kg/m^2^)	23.82 (3.37)/16.41–36.05	23.87 (3.39)/16.41–36.05
Hypertension (yes)	51 (36.7)	44 (34.9)
Diabetes mellitus (yes)	24 (17.3)	21 (16.7)
Heart disease (yes)	14 (10.1)	13 (10.3)
Renal disease (yes)	5 (3.6)	5 (4.0)
Stroke (yes)	8 (5.8)	6 (4.8)
Dementia (yes)	4 (2.9)	3 (2.4)
Depression (yes)	5 (3.6)	5 (4.0)
Cancer (yes)	15 (10.8)	13 (10.3)

*Baseline measures were examined from April, 20, 2020, to May 15, 2020; follow-up measures were examined from May, 18, 2021, to June 14, 2021*.

Table 2 presents the participants' psychological and behavioral changes. The participants exhibited significantly increased fear of COVID-19 (*d* = 0.39, *p* < 0.001), trust in COVID-19 information (*d* = 0.76, *p* < 0.001), COVID-19 news searching behavior (*d* = 0.75, *p* < 0.001), and perceived infectability (*d* = 0.51, *p* < 0.001) in the follow-up assessment than they did in the baseline assessment. By contrast, the participants had significantly reduced their preventive COVID-19 behaviors (*d* = 0.63, *p* < 0.001) and paying attention to COVID-19 news (*d* = 0.25, *p* = 0.007).

**Table 2:** Psychological and behavioral changes before and after community outbreak (*n* = 126).

	***Mean*** **(*****SD*****)**		***t* (*p*-value)**	**Cohen's *d***
	**Baseline**	**Follow-up**		
Fear of COVID-19	1.81 (0.82)	2.16 (0.63)	4.20 (<0.001)	0.39
Trust in COVID-19 information	2.94 (0.53)	3.41 (0.39)	8.49 (<0.001)	0.76
Paying attention to COVID-19 news	4.18 (1.04)	3.89 (0.69)	2.75 (0.007)	0.25
Preventive COVID-19 behaviors	4.79 (0.53)	4.36 (0.57)	7.09 (<0.001)	0.63
COVID-19 news searching behavior	1.99 (1.42)	3.16 (0.98)	8.29 (<0.001)	0.75
Perceived infectability	1.54 (0.90)	2.16 (0.90)	5.74 (<0.001)	0.51

*Baseline measures were examined from April 20, 2020, to May 15, 2020; follow-up measures were examined from May 18, 2021, to June 14, 2021*.

* community outbreak started in mid-May in Taiwan*.

Table 3 lists the correlation coefficients between the studied variables at two time points (i.e., before and after the community outbreak in Taiwan). Among the variables assessed before the community outbreak, paying attention to COVID-19 news was significantly associated with trust in COVID-19 information (*r* = 0.28, *p* < 0.01) and COVID-19 news searching behavior (*r* = 0.23, *p* < 0.01); preventive COVID-19 behaviors were significantly associated with perceived infectability (*r* = 0.40, *p* < 0.001) and COVID-19 news searching behavior (*r* = −0.17, *p* < 0.05); and COVID-19 news searching behavior was significantly associated with perceived infectability (*r* = 0.28, *p* < 0.01). Among the variables assessed after the community outbreak, fear of COVID-19 was significantly associated with paying attention to COVID-19 news, preventive COVID-19 behaviors, COVID-19 news searching behavior, and perceived infectability (*r* = 0.25 to 0.49, *p* < 0.01), and trust in COVID-19 information was significantly associated with preventive COVID-19 behaviors (*r* = 0.30, *p* < 0.01).

**Table 3:** Correlation coefficients between studied variables before (*n* = 139) and after (*n* = 126) the community outbreak.

	**1**	**2**	**3**	**4**	**5**	**6**	**7**	**8**	**9**	**10**
1. Age	–	0.11	−0.36^***^	−0.03	0.16	−0.19^*^	−0.06	−0.06	−0.27^**^	0.19^*^
2. Sex	0.11	–	0.16	0.14	0.08	0.04	0.11	0.03	0.17	0.04
3. Education	−0.36^***^	0.16	–	−0.04	0.05	0.23^*^	0.15	0.15	0.15	−0.06
4. Body mass index	−0.03	0.14	−0.04	–	0.07	−0.02	0.02	0.09	−0.03	0.04
5. Fear	0.11	0.16	−0.04	0.05	–	0.12	0.42^***^	0.32^***^	0.25^**^	0.49^***^
6. Trust	0.02	0.01	−0.06	−0.06	0.15	–	0.01	0.30^**^	0.17	−0.09
7. Attention	−0.13	0.03	0.19^*^	0.04	0.01	0.28^**^	–	0.21^*^	0.32^***^	0.34^***^
8. Preventive behavior	−0.07	−0.18^*^	−0.05	0.03	−0.07	−0.04	−0.02	–	0.09	−0.01
9. Search	−0.25^***^	0.03	0.25^*^	0.11	0.04	0.15	0.23^**^	−0.17^*^	–	0.29^**^
10. Infectability	−0.09	0.06	0.04	−0.09	0.40^***^	0.04	0.10	−0.14	0.28^**^	–

*Sex (male vs. female) and education (junior high or below vs. senior high or above) were treated as dichotomous in the correlation matrix*.

*Fear, fear of COVID-19; Trust, trust in COVID-19 information; Attention, paying attention to COVID-19 news; Preventive behavior, preventive COVID-19 behaviors; Search, COVID-19 news searching behavior; Infectability, perceived infectability*.

*Coefficients in the lower triangular matrix are baseline measures examined from April 20, 2020, to May 15, 2020; coefficients in the upper triangular matrix are follow-up measures examined from May 18, 2021, to June 14, 2021*.

*A community outbreak started in mid-May in Taiwan*.

* *p < 0.05;*

** *p < 0.01;*

*** *p < 0.001*.

**Figure 3:**
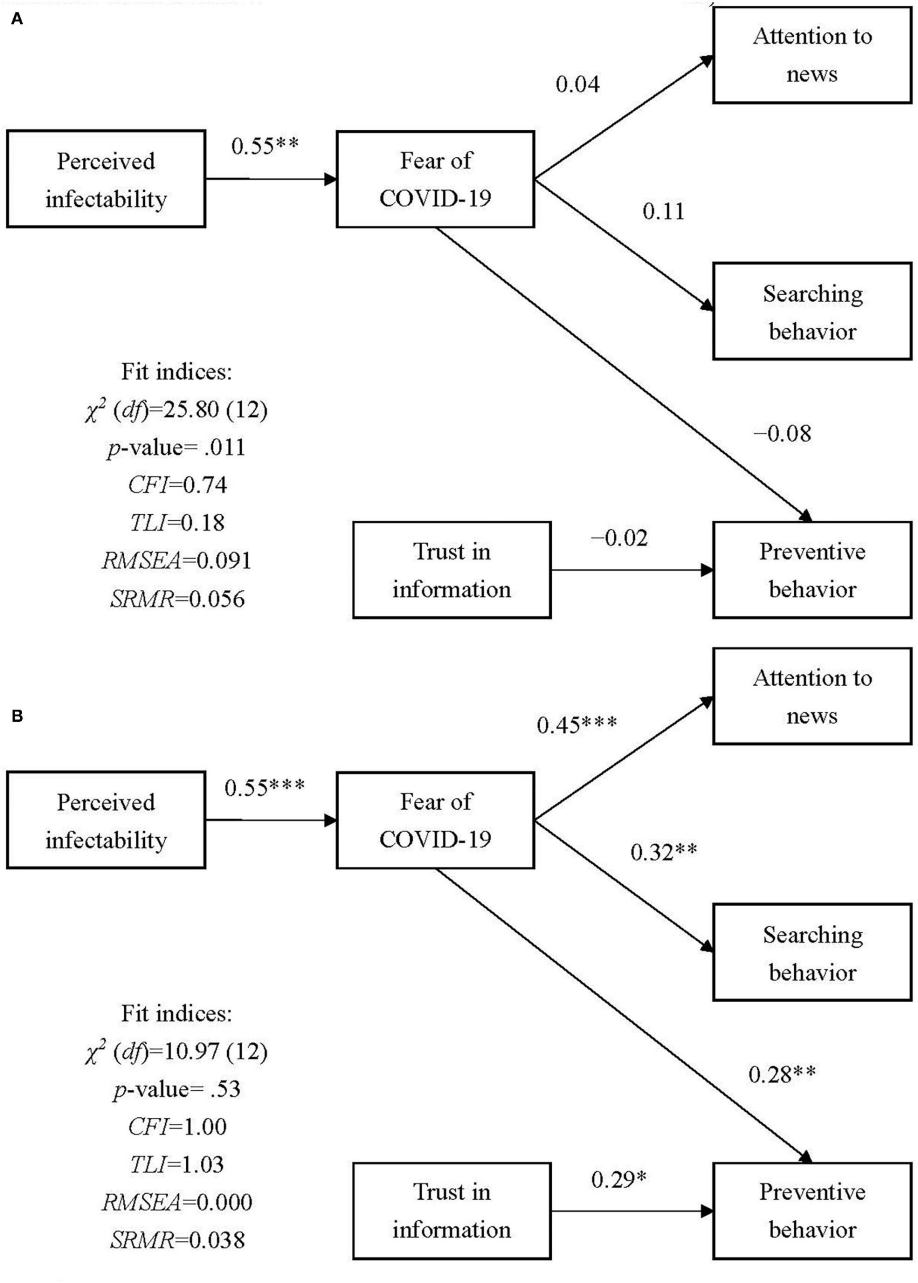
Results of the proposed model at two time points. Age, sex, education, body mass index, and comorbidities were controlled in the model. **(A)** Results from baseline measures examined before the outbreak (April 20, 2020, to May 15, 2020); **(B)** Results from follow-up measures assessed after the outbreak (May 18, 2021, to June 14, 2021). *p < 0.05; **p < 0.01; ***p < 0.001.

The proposed model in the present study was not supported using baseline data (*p* of χ^2^ test = 0.011, CFI = 0.74, TLI = 0.18, RMSEA = 0.091, SRMR = 0.056). The path coefficients were not significant except for the path between perceived infectability and fear of COVID-19 in the proposed model when it was fitted with baseline data (Figure 3A). However, the proposed model showed a satisfactory fit with follow-up data (*p* of χ^2^ test = 0.53, CFI = 1.00, TLI = 1.03, RMSEA = 0.000, SRMR = 0.038). Moreover, all path coefficients (i.e., perceived infectability to fear of COVID-19; fear of COVID-19 to paying attention to COVID-19 news, COVID-19 news searching behavior, and preventive COVID-19 behaviors; and trust in COVID-19 information to preventive COVID-19 behaviors) were significant in the proposed model when it was fitted with follow-up data (Figure 3B).

## Discussion

To the best of our knowledge, the present study is the first to examine associations among perceived infectability, fear of COVID-19, trust in COVID-19 information, and COVID-19-related behaviors (i.e., paying attention to COVID-19 news, COVID-19 news searching behavior, and preventive COVID-19 behaviors) in two extremely different levels of COVID-19 severity among older people. The results of the present study showed that perceived infectability was significantly and positively associated with the fear of COVID-19. Moreover, fear of COVID-19 was positively associated with COVID-19-related behaviors during the period of severe COVID-19 community outbreak but not during the period of mild COVID-19 community outbreak. Similarly, trust in COVID-19 information was positively associated with preventive COVID-19 behaviors during the period of severe COVID-19 community outbreak but not during the period of mild COVID-19 community outbreak. The findings supported all the hypotheses for the period with severe COVID-19 community outbreak but only Hypothesis 1 for the period with mild COVID-19 community outbreak. Moreover, increased levels of fear of COVID-19, trust in COVID-19 information, COVID-19 news searching behavior, and perceived infectability were observed during the period of severe COVID-19 community outbreak. By contrast, the levels of paying attention to COVID-19 news and preventive COVID-19 behaviors decreased during the period of severe COVID-19 community outbreak.

The increased levels of perceived infectability, fear of COVID-19, trust in COVID-19 information, and COVID-19 news searching behavior can be explained by the increased severity of the COVID-19 outbreak in Taiwan. The substantial increase in confirmed COVID-19 cases and deaths during May and June 2021 (confirmed cases increased from 1,132 on May 1, 2021 to 10,846 on June 14, 2021; deaths increased from 12 to 438 during this period) likely scared residents, especially older people who can develop severe health problems due to COVID-19 infection (18, 19). By contrast, decreased levels of preventive behaviors and paying attention to COVID-19 news were observed among older people. This finding may be explained by behavioral fatigue (46, 47). The present sample showed relatively high scores in paying attention to COVID-19 news (4.18 of 5) and preventive COVID-19 behaviors (4.79 of 5) during the baseline survey. Therefore, they might have not been able to maintain such high levels of behavior over a year. Future studies should explore the effects of and mechanisms underlying behavioral fatigue to assist older people in maintaining their preventive behaviors.

The proposed model with five hypotheses was supported by data collected at follow-up but not by data collected at baseline. This finding indicates that prior theories (e.g., the health belief model, protection motivation model, and fear drive model) (28–32) could be used to explain COVID-19 prevention among older people. Preventive behaviors may not be induced or triggered when older people's fear is low. Prior evidence regarding the effectiveness of fear on individuals' preventive COVID-19 behaviors depends on relatively high levels of fear (5, 6, 9, 34). Moreover, the follow-up data showed a higher level of fear than the baseline data. Therefore, the findings suggest that older people should have some level of fear to engage in preventive behaviors. However, additional empirical evidence is required to corroborate such a postulation.

The findings of the present study has some implications. First, healthcare providers and policymakers should develop potential strategies to prevent older people from feeling fatigue in practicing preventive behaviors. It will be a long time before the COVID-19 pandemic is fully under control. Given the COVID-19 pandemic may last for a long period, maintaining preventive COVID-19 behaviors will be crucial before the pandemic is under control. Therefore, strategies are needed to prevent behavioral fatigue among older people. Second, healthcare providers and policymakers should monitor the fear levels among older citizens and apply fear appeals when necessary to increase their engagement in preventive behaviors (48–50). More specifically, fear appeals emphasize the potential danger and harm for health and may facilitate the adherence of preventive COVID-19 behaviors among older people (50, 51) although it should be noted that fear may increase due to the information provided (52, 53). Therefore, when fear is low, the government should disseminate accurate information regarding the negative consequences of becoming infected with COVID-19 to increase older people's awareness and attention to aid compliance and facilitate preventive behaviors. However, such information should be accompanied by information stating that preventive COVID-19 behaviors can significantly decrease their chances of getting COVID-19 infection which would minimize panic and/or high levels of psychological distress. Moreover, the government may need to implement a mental health campaign to help older people cope with their psychological distress concerning the potentially fatal consequences of COVID-19. Another implication of the study relates to planning health education interventions using fear-based theories/model to increase observance of preventive strategies among older adults. Indeed, since the level of fear and the sense of infectability may be significantly be associated with adherence to preventive measures, based on the present study's findings, using such interventions may include positive impacts on behavioral compliance in this population.

This study has some limitations that should be addressed. First, the modest number of recruited participants were all older outpatients from a medical center, indicating that they had health-seeking behaviors. Therefore, the participants might have had a higher level of engagement in preventive behaviors during the pandemic. Consequently, the present findings cannot be generalized to all older people, especially those who do not have health-seeking behavior. Second, the measures were assessed using different methods in the two surveys. The baseline survey was conducted utilizing in-person interviews whereas the follow-up survey was conducted utilizing telephonic interviews. However, given that the period of the follow-up survey was during a severe community outbreak and unnecessary in-person contact was prohibited, a telephone interview was one of the few methods that could be adopted to obtain survey data. Nevertheless, the present study used the same interviewers to administer both surveys to reduce potential rater bias during the two periods of data collection. Third, some psychological constructs in the present study were assessed using a single-item measure. These constructs may have poorer psychometric properties (e.g., low internal validity and reliability with inconsistent outcomes between the baseline and follow-up measures in the present study) than those assessed using multiple items (54). However, given that the target population in the present study was older people seeking medical services, a practical and strategic decision was taken by the research team to reduce the length of the survey to minimize the burden for this specific population. Finally, the preventive COVID-19 behaviors assessed in the present study may no longer be the best preventive indicators (e.g., many governments have suggested wearing a mask to be a good preventive COVID-19 behavior; also, the development of effective vaccines means that vaccination uptake is an important preventive COVID-19 behavior (55); however, these were not assessed in the present study). Therefore, in order to align the follow-up measures with the baseline measures, the present study did not include these latest preventive behaviors for data analyses.

## Conclusion

The findings of the present study demonstrated that the severity of a COVID-19 outbreak may alter older people's psychological status and related behaviors. Fear of COVID-19, trust in COVID-19 information, and COVID-19 news searching behavior were particularly increased. However, because of behavioral fatigue, older people's preventive COVID-19 behaviors and their paying attention to COVID-19 news were decreased in the severe COVID-19 outbreak period. Moreover, the association between preventive COVID-19 behaviors and fear of COVID-19 was observed only when the older people had some level of fear. Based on the findings, governments and healthcare providers may need to develop efficient methods specifically for older people to prevent behavioral fatigue until the pandemic is fully controlled. Replication of this study in a situation that older adults are vaccinated against COVID-19 throughout the community may indicate how such controlling strategies may affect the process of interaction between fear and behavioral aspects of prevention in this population.

## Data Availability Statement

The raw data supporting the conclusions of this article will be made available by the authors, without undue reservation.

## Ethics Statement

This study was approved by the Ethical Committee of Taipei Medical University (registered number: TMU-JIRB N202005044). The patients/participants provided their written informed consent to participate in this study.

## Author Contributions

Y-JK, Y-PC, H-WW, C-hL, CS, N-YK, and C-YL contributed to the conception and design of the study. Y-JK, Y-PC, CS, N-YK, and C-YL organized the database. H-WW, MS, and C-YL performed the statistical analysis. Y-JK, Y-PC, H-WW, C-hL, and MS interpreted the results. Y-JK, Y-PC, and C-YL wrote the first draft of the manuscript. MS wrote sections of the manuscript. H-WW, C-hL, CS, N-YK, MG, and C-YL critically reviewed the manuscript. MG edited the final revised version. All authors contributed to manuscript revision, read and approved the submitted version.

## Funding

This study was supported in part by the Ministry of Science and Technology, Taiwan (grant number: MOST109-2327-B-006-005) and in part by the Taipei Municipal Wanfang Hospital Cross-Institutions Fund (grant number: 110-swf-01).

## Conflict of Interest

The authors declare that the research was conducted in the absence of any commercial or financial relationships that could be construed as a potential conflict of interest.

## Publisher's Note

All claims expressed in this article are solely those of the authors and do not necessarily represent those of their affiliated organizations, or those of the publisher, the editors and the reviewers. Any product that may be evaluated in this article, or claim that may be made by its manufacturer, is not guaranteed or endorsed by the publisher.

## References

[1] Alimoradi Z Broström A Tsang HW Griffiths MD Haghayegh S Ohayon MM. Sleep problems during COVID-19 pandemic and its' association to psychological distress: a systematic review and meta-analysis. EClinicalMedicine. (2021) 36:100916. 10.1016/j.eclinm.2021.100916 34131640 PMC8192091

[2] Alimoradi Z Gozal D Tsang HW Lin CY Broström A Ohayon MM. Gender-specific estimates of sleep problems during the COVID-19 pandemic: Systematic review and meta-analysis. J Sleep Res. (2021). 10.1111/jsr.13432. [Epub ahead of print]. 34245055 PMC8420603

[3] Malik S Ullah I Irfan M Ahorsu DK Lin C-Y Pakpour AH. Fear of COVID-19 and workplace phobia among Pakistani doctors: a survey study. BMC Public Health. (2021) 21:833. 10.1186/s12889-021-10873-y 33931040 PMC8086971

[4] Mamun MA Sakib N Gozal D Bhuiyan AI Hossain S Bodrud-Doza M. The COVID-19 pandemic and serious psychological consequences in Bangladesh: a population-based nationwide study. J Affect Disord. (2021) 279:462–72. 10.1016/j.jad.2020.10.036 33120247 PMC7568472

[5] Ahorsu DK Lin C-Y Pakpour AH. The association between health status and insomnia, mental health, and preventive behaviors: the mediating role of fear of COVID-19. Gerontol Geriatr Med. (2020) 6:2333721420966081. 10.1177/2333721420966081 33195740 PMC7594224

[6] Lin CY Imani V Majd NR Ghasemi Z Griffiths MD Hamilton K. Using an integrated social cognition model to predict COVID-19 preventive behaviours. Br J Health Psychol. (2020) 25:981–1005. 10.1111/bjhp.12465 32780891 PMC7436576

[7] Chen I-H Ahorsu DK Ko N-Y Yen C-F Lin C-Y Griffiths MD. Adapting the motors of Influenza vaccination acceptance scale into the motors of COVID-19 vaccination acceptance scale: psychometric evaluation among Mainland Chinese university students. Vaccine. (2021) 39:4510–5. 10.1016/j.vaccine.2021.06.044 34217571 PMC8216877

[8] Fan C-W Chen I-H Ko N-Y Yen C-F Lin C-Y Griffiths MD. Extended theory of planned behavior in explaining the intention to COVID-19 vaccination uptake among mainland Chinese university students: an online survey study. Hum Vaccines Immunother. (2021) 17:3413–20. 10.1080/21645515.2021.1933687 34170792 PMC8437493

[9] Yahaghi R Ahmadizade S Fotuhi R Taherkhani E Ranjbaran M Buchali Z. Fear of COVID-19 and perceived COVID-19 infectability supplement theory of planned behavior to explain Iranians' intention to get COVID-19 vaccinated. Vaccines. (2021) 9:684. 10.3390/vaccines9070684 34206226 PMC8310138

[10] Mao L. Evaluating the combined effectiveness of influenza control strategies and human preventive behavior. PLoS One. (2011) 6:e24706. 10.1371/journal.pone.0024706 22043275 PMC3197180

[11] Shaw R Kim Y-K Hua J. Governance, technology and citizen behavior in pandemic: Lessons from COVID-19 in East Asia. Progr Disaster Sci. (2020) 6:100090. 10.1016/j.pdisas.2020.100090 34171010 PMC7194878

[12] Sim SW Moey KSP Tan NC. The use of facemasks to prevent respiratory infection: a literature review in the context of the Health Belief Model. Singapore Med J. (2014) 55:160. 10.11622/smedj.2014037 24664384 PMC4293989

[13] Abel T McQueen D. The COVID-19 pandemic calls for spatial distancing and social closeness: not for social distancing! *Int J Public Health*. (2020) 65:231. 10.1007/s00038-020-01366-7 32239256 PMC7111296

[14] Chen CY Chen IH Hou WL Potenza MN O'Brien KS Lin CY. The relationship between children's problematic internet-related behaviors and psychological distress during the onset of the covid-19 pandemic: a longitudinal study. J Addict Med. (2021). 10.1097/ADM.0000000000000845. [Epub ahead of print]. 33770003 PMC8919938

[15] Fung XC Siu AM Potenza MN O'brien KS Latner JD Chen C-Y. Problematic use of internet-related activities and perceived weight stigma in schoolchildren: A longitudinal study across different epidemic periods of COVID-19 in China. Front Psychiatr. (2021) 12:675839. 10.3389/fpsyt.2021.675839 34108898 PMC8183469

[16] Lin CY Hou WL Mamun MA Aparecido Da Silva J Broche-Pérez Y Ullah I. Fear of COVID-19 Scale (FCV-19S) across countries: measurement invariance issues. Nurs Open. (2021) 8:1892–908. 10.1002/nop2.855 33745219 PMC8186712

[17] Wang CJ Ng CY Brook RH. Response to COVID-19 in Taiwan: big data analytics, new technology, and proactive testing. JAMA. (2020) 323:1341–2. 10.1001/jama.2020.3151 32125371

[18] Moccia F Gerbino A Lionetti V Miragoli M Munaron L Pagliaro P. COVID-19-associated cardiovascular morbidity in older adults: a position paper from the Italian Society of Cardiovascular Researches. Geroscience. (2020) 42:1021–49. 10.1007/s11357-020-00198-w 32430627 PMC7237344

[19] Dariya B Nagaraju GP. Understanding novel COVID-19: its impact on organ failure and risk assessment for diabetic and cancer patients. Cytokine Growth Factor Rev. (2020) 53:43–52. 10.1016/j.cytogfr.2020.05.001 32409230 PMC7202812

[20] Simonnet A Chetboun M Poissy J Raverdy V Noulette J Duhamel A. High prevalence of obesity in severe acute respiratory syndrome coronavirus-2 (SARS-CoV-2) requiring invasive mechanical ventilation. Obesity. (2020) 28:1195–9. 10.1002/oby.22831 32271993 PMC7262326

[21] Wang D Hu B Hu C Zhu F Liu X Zhang J. Clinical characteristics of 138 hospitalized patients with 2019 novel coronavirus–infected pneumonia in Wuhan, China. JAMA. (2020) 323:1061–9. 10.1001/jama.2020.1585 32031570 PMC7042881

[22] Xiao H Zhang Y Kong D Li S Yang N. Social capital and sleep quality in individuals who self-isolated for 14 days during the coronavirus disease 2019 (COVID-19) outbreak in January 2020 in China. Med Sci Monitor. (2020) 26:e923921. 10.12659/MSM.923921 32194290 PMC7111105

[23] Chen C-Y Chen I-H O'brien KS Latner JD Lin C-Y. Psychological distress and internet-related behaviors between schoolchildren with and without overweight during the COVID-19 outbreak. Int J Obes. (2021) 45:677–86. 10.1038/s41366-021-00741-5 33495523 PMC7829481

[24] Li YP Lin CY Hu FW Shih SA. Short versions of the Geriatric Depression Scale (GDS) among widowed older people in Taiwan: comparing their psychometric properties. Aust J Ageing. (2021). 10.1111/ajag.12942. [Epub ahead of print]. 33724655

[25] Serby M Yu M. Overview: depression in the elderly. Mt Sinai J Med. (2003) 70:38–44. 12516008

[26] Chen Y-P Wang S-M Wu Y Lin H-Y Wu C-C Chuang T-Y. Worsen depression after viscosupplementation treatment for geriatric people with knee osteoarthritis? Int J Clin Health Psychol. (2019) 19:31–40. 10.1016/j.ijchp.2018.10.001 30619495 PMC6300725

[27] Moussavi S Chatterji S Verdes E Tandon A Patel V Ustun B. Depression, chronic diseases, and decrements in health: results from the World Health Surveys. Lancet. (2007) 370:851–8. 10.1016/S0140-6736(07)61415-9 17826170

[28] Chang KC Strong C Pakpour AH Griffiths MD Lin C Y. Factors related to preventive COVID-19 infection behaviors among people with mental illness. J Formosan Med Assoc. (2020) 119:1772–80. 10.1016/j.jfma.2020.07.032 32773260 PMC7388748

[29] Green EC Murphy E. (2014). “Health belief model,” in The Wiley Blackwell Encyclopedia of Health, Illness, Behavior, and Society, eds W. C. Cockerham, R. Dingwall, and S. R. Quah (New York, NY: Wiley), 766–9. 10.1002/9781118410868.wbehibs410

[30] Leventhal H Safer MA Panagis DM. The impact of communications on the self-regulation of health beliefs, decisions, and behavior. Health Educ Q. (1983) 10:3–29. 10.1177/109019818301000101 6629788

[31] Pakpour AH Griffiths MD. The fear of COVID-19 and its role in preventive behaviors. J Concurr Disord. (2020) 2:58–63.34583603

[32] Rogers RW. A protection motivation theory of fear appeals and attitude change1. J Psychol. (1975) 91:93–114. 10.1080/00223980.1975.9915803 28136248

[33] Wang P-W Ahorsu DK Lin C-Y Chen I-H Yen C-F Kuo Y-J. Motivation to have Covid-19 vaccination explained using an extended protection motivation theory among university students in china: The role of information sources. Vaccines. (2021) 9:380. 10.3390/vaccines9040380 33924604 PMC8070343

[34] Wong LP Alias H Wong P-F Lee HY Abubakar S. The use of the health belief model to assess predictors of intent to receive the COVID-19 vaccine and willingness to pay. Hum Vaccines Immunother. (2020) 16:2204–14. 10.1080/21645515.2020.1790279 32730103 PMC7553708

[35] Basch CH Hillyer GC Meleo-Erwin ZC Jaime C Mohlman J Basch CE. Preventive behaviors conveyed on YouTube to mitigate transmission of COVID-19: cross-sectional study. JMIR Public Health Surveil. (2020) 6:e18807. 10.2196/18807 32240096 PMC7124952

[36] Pramukti I Strong C Sitthimongkol Y Setiawan A Pandin MGR Yen C-F. Anxiety and suicidal thoughts during the COVID-19 pandemic: cross-country comparative study among Indonesian, Taiwanese, and Thai University students. J Med Internet Res. (2020) 22:e24487. 10.2196/24487 33296867 PMC7772053

[37] Lu M-Y Ahorsu DK Kukreti S Strong C Lin Y-H Kuo Y-J. The prevalence of post-traumatic stress disorder symptoms, sleep problems, and psychological distress among COVID-19 frontline Healthcare Workers in Taiwan. Front Psychiatr. (2021) 12:705657. 10.3389/fpsyt.2021.705657 34322044 PMC8312888

[38] Chang K-C Hou W-L Pakpour AH Lin C-Y Griffiths MD. Psychometric testing of three COVID-19-related scales among people with mental illness. Int J Ment Health Addict. (2020). 10.1007/s11469-020-00361-6. [Epub ahead of print]. 32837442 PMC7354353

[39] Ahorsu DK Lin C-Y Imani V Saffari M Griffiths MD Pakpour AH. The fear of COVID-19 scale: development and initial validation. Int J Ment Health Addict. (2020). 10.1007/s11469-020-00270-8. [Epub ahead of print]. 32226353 PMC7100496

[40] Cohn J. Statistical Power Analysis for the Behavioral Sciences. Hillsdale, NJ: Lawrence Earlbam Associates (1988).

[41] Kohler H Bäuerle A Schweda A Weismüller B Fink M Musche V. Increased COVID-19-related fear and subjective risk perception regarding COVID-19 affects behavior in individuals with internal high-risk diseases. J Prim Care Commun Health. (2021) 12:2150132721996898. 10.1177/2150132721996898 33719697 PMC8851367

[42] Niño M Harris C Drawve G Fitzpatrick KM. Race and ethnicity, gender, and age on perceived threats and fear of COVID-19: Evidence from two national data sources. SSM Popul Health. (2021) 13:100717. 10.1016/j.ssmph.2020.100717 33344747 PMC7733547

[43] Lin Y-C Fung XC Tsai M-C Strong C Hsieh Y-P Lin C-Y. Insufficient physical activity and overweight: does caregiver screen-viewing matter? J Child Fam Stud. (2019) 28:286–97. 10.1007/s10826-018-1247-5

[44] Yam C-W Pakpour AH Griffiths MD Yau W-Y Lo C-LM Ng JM. Psychometric testing of three Chinese online-related addictive behavior instruments among Hong Kong university students. Psychiatr Q. (2019) 90:117–28. 10.1007/s11126-018-9610-7 30328020

[45] Rosseel Y. Lavaan: an R package for structural equation modeling and more. Version 0.5–12 (BETA). J Stat Softw. (2012) 48:1–36. 10.18637/jss.v048.i02

[46] Abbasi K. Behavioural fatigue: a flawed idea central to a flawed pandemic response. Br Med J. (2020) 370:m3093. 10.1136/bmj.m309328850992

[47] Harvey N. Behavioral fatigue: real phenomenon, naïve construct, or policy contrivance? Front Psychol. (2020) 11:2960. 10.3389/fpsyg.2020.589892 33224078 PMC7674166

[48] Dillard JP Plotnick CA Godbold LC Freimuth VS Edgar T. The multiple affective outcomes of AIDS PSAs: fear appeals do more than scare people. Commun Res. (1996) 23:44–72. 10.1177/009365096023001002

[49] Maddux JE Rogers RW. Protection motivation and self-efficacy: a revised theory of fear appeals and attitude change. J Exp Soc Psychol. (1983) 19:469–79. 10.1016/0022-1031(83)90023-9

[50] Tannenbaum MB Hepler J Zimmerman RS Saul L Jacobs S Wilson K. Appealing to fear: a meta-analysis of fear appeal effectiveness and theories. Psychol Bull. (2015) 141:1178–204. 10.1037/a0039729 26501228 PMC5789790

[51] Simpson JK. Appeal to fear in health care: appropriate or inappropriate? Chiropr Manual Ther. (2017) 25:27. 10.1186/s12998-017-0157-8 28932388 PMC5605990

[52] Liu H Liu W Yoganathan V Osburg V-S. COVID-19 information overload and generation Z's social media discontinuance intention during the pandemic lockdown. Technol Forecast Soc Change. (2021) 166:120600. 10.1016/j.techfore.2021.120600 34876758 PMC8640972

[53] Steele H. COVID-19, fear and the future: an attachment perspective. Clin Neuropsychiatr. (2020) 2:97–9. 10.36131/CN20200213 PMC8629047 34908977

[54] Fisher GG Matthews RA Gibbons AM. Developing and investigating the use of single-item measures in organizational research. J Occup Health Psychol. (2016) 21:3. 10.1037/a0039139 25894198

[55] National Center for High-performance Computing in Taiwan. COVID-19 Figure in Taiwan. (2021). Available online at: https://covid-19.nchc.org.tw/city_confirmed.php?mycity=%E5%8F%B0%E5%8C%97%E5%B8%82 (accessed September 15, 2021).

